# Sinhyotaklisan as a Potential Therapeutic for Psoriasis: Network Pharmacology and Experimental Validation

**DOI:** 10.3390/ijms26115082

**Published:** 2025-05-25

**Authors:** Jung-Yun Ahn, Dong-Woo Lim, Jin-Hee Kim, Sung-Yun Park, Sun-Dong Park, Ju-Hee Lee

**Affiliations:** 1 College of Korean Medicine, Dongguk University, Goyang 10326, Republic of Korea; rurujy77@naver.com (J.-Y.A.); greatwoodong@dongguk.edu (D.-W.L.); bmepark@dongguk.ac.kr (S.-Y.P.); 2 Department of Biomedical Laboratory Science, College of Health Science, Cheongju University, Cheongju 28503, Republic of Korea; jinheekim@cju.ac.kr

**Keywords:** Sinhyotaklisan, psoriasis, network pharmacology, enrichment analysis, angiogenesis

## Abstract

Sinhyotaklisan (SHTLS) is a traditional herbal prescription composed of *Lonicerae Flos*, *Angelicae Gigantis Radix*, *Astragali Radix*, and *Glycyrrhizae Radix et Rhizoma*, commonly used to treat skin disorders. This study aimed to investigate the therapeutic effects and underlying mechanisms of SHTLS in psoriasis through the network pharmacology analysis and experimental validation in vitro and in vivo. Bioactive compounds and molecular targets were identified using the Traditional Chinese Medicine Systems Pharmacology database, and key protein–protein interaction networks were analyzed via STRING and Cytoscape. In vitro, HaCaT cells were pretreated with SHTLS and stimulated with TNF-α, followed by assessments using proliferation assays, scratch assays, quantitative real-time PCR, and Western blotting. In vivo, the anti-psoriatic effects of SHTLS were evaluated in an imiquimod-induced psoriatic mouse model. A total of 36 key targets were significantly enriched in TNF-α, MAPK, HIF-1α, and IL-17 signaling pathways. SHTLS suppressed TNF-α-induced expression of VEGF and HIF-1α, while upregulating p53, thereby inhibiting keratinocyte hyperproliferation and angiogenesis. It also reduced IL-6 and IL-8 levels and blocked activation of the NF-κB and MAPK pathways. Histological analysis confirmed that SHTLS alleviated psoriatic lesions in vivo. These findings suggest that SHTLS may be a promising therapeutic candidate for psoriasis by targeting hyperproliferation, angiogenesis, and inflammation.

## 1. Introduction

Psoriasis is a chronic autoimmune inflammatory skin disorder characterized by red, scaly skin, dryness, itching, and thick whitish-silver plaques [1]. Globally, approximately 2~3% of the population is afflicted by psoriasis, and its prevalence is expected to rise [2,3,4]. The main features of psoriatic lesions include chronic inflammatory responses, increased angiogenesis, hyperkeratosis, and abnormal keratinocyte differentiation [5]. Common clinical characteristics of psoriasis include erythema, epidermal hyperplasia, and scaling papules and plaques [6]. Therefore, previous pharmacological research aimed at developing new psoriatic treatments has focused on targeting these pathological features and preventing lesion development [7].

As a result of intrinsic or extrinsic triggers, a complex cascade of inflammatory responses is activated in psoriatic skin lesions through stimulation by pro-inflammatory cytokines such as tumor necrosis factor-α (TNF-α), interleukin-8 (IL-8), and interleukin-6 (IL-6) [8]. In this cascade, reactive oxygen species (ROS)-sensitive signaling pathways, such as the mitogen-activated protein kinase (MAPK) and nuclear factor-kappa B (NF-κB) signaling pathway, are activated [9,10]. The chronic and recurrent nature of these inflammatory responses can lead to secondary infection [11]. Thus, effective management of psoriasis requires adequate modulation of refractory inflammation by targeting abnormal immune responses and the involved immune cells [12].

At the cellular level, psoriatic skin cells exhibit an increased turnover rate and hyperproliferation of keratinocytes despite the increased TNF-α level [13]. This paradox is partially explained by NF-κB activation, which inhibits TNF-α-induced apoptosis [14]. Compounds that inhibit NF-κB pathway activation could be useful in controlling hyperproliferation of human keratinocytes [15]. Furthermore, IL-8 has been implicated in promoting cell survival and proliferation in various human cell lines, including tumor cells, endothelial cells, and epidermal keratinocytes [16,17]. These findings suggest that IL-8 and NF-κB play critical roles in keratinocyte proliferation and may serve as therapeutic targets.

Pathological angiogenesis, characterized by the formation of new blood vessels from pre-existing ones, is a key feature of inflammatory diseases [18,19]. According to substantial evidence, vascular endothelial growth factor (VEGF) is overexpressed in psoriatic keratinocytes, and its serum levels correlate with psoriasis severity, as measured by the Psoriasis Area and Severity Index (PASI) [18,20,21]. Therefore, VEGF-targeted treatment strategies could help prevent psoriatic progress.

Topical corticosteroids are widely used as a first-line treatment for psoriasis, either locally or systematically [22,23]. Although steroidal medications may provide temporary relief, long-term use is associated with side effects and drug tolerance [23]. The adverse effects of topical corticosteroids include skin atrophy, striae, folliculitis, telangiectasia, and purpura, while systemic corticosteroids, though less frequently used, can cause serious complications such as Cushing’s syndrome, diabetes, and adrenal suppression [24]. In addition, psoriasis is a complex disease with multifaceted pathogenesis and mechanisms, making it difficult to address with a single drug. In this regard, traditional herbal medicine presents a promising alternative due to its natural origin and multi-compound, multi-target properties.

Korean medicine follows a specialized theory and treatment approach that utilizes various herbs to treat inflammatory skin diseases. Sinhyotaklisan (SHTLS) is a renowned herbal prescription composed of four medicinal herbs: *Lonicerae Flos* (LF; *Lonicera japonica* Thunb.), *Glycyrrhizae Radix et Rhizoma* (GR; *Glycyrrhiza uralensis* Fisch. ex DC.), *Astragali Radix* (ASR; *Astragalus membranaceus* Fisch. ex Bunge), and *Angelicae Gigantis Radix* (ANR; *Angelica gigas* Nakai) [25]. In a previous study, we investigated the antioxidant, anti-bacterial, and anti-inflammatory effects of SHTLS in RAW 264.7 cells, suggesting that the regulation of the NF-κB and MAPK pathways plays a major role in its mechanism of action [26]. Moreover, SHTLS demonstrated significant ROS-scavenging effects and enhanced intracellular ROS defense mechanisms, such as heme oxygenase-1 (HO-1). Inhibition of HO-1 undermined the effects of SHTLS, further supporting its role in oxidative stress regulation. Given that SHTLS has been traditionally used for various inflammatory skin conditions [25], its potential efficacy in treating psoriatic lesions is promising and warrants further investigation.

Network pharmacology, a valuable strategy for studying the complex therapeutic networks of herbal treatments, can enhance our understanding of the mechanisms underlying herbal prescriptions for psoriasis. Some researchers have already applied the network pharmacology analysis to investigate their effects [27,28]. A recent study on *Qingre Lishi Decoction* in TNF-α-treated HaCaT cells found that its anti-psoriatic effects were associated with reduced cytokine production and suppression of NF-κB activation [29].

In the present study, we analyzed and predicted the potential targets and mechanisms of SHTLS in psoriasis treatment, using network pharmacological methods. Considering two main psoriatic features, enhanced angiogenesis and hyperproliferation of keratinocytes, we investigated the multi-target efficacy and mechanisms of SHTLS in TNF-α-treated HaCaT cells in vitro. Furthermore, we evaluated the effectiveness of SHTLS in an in vivo model of imiquimod (IMQ)-induced psoriatic skin lesions.

## 2. Results

### 2.1. Screening of Potential Compounds and Key Targets of Sinhyotaklisan (SHTLS) in Psoriasis

**Figure 1:**
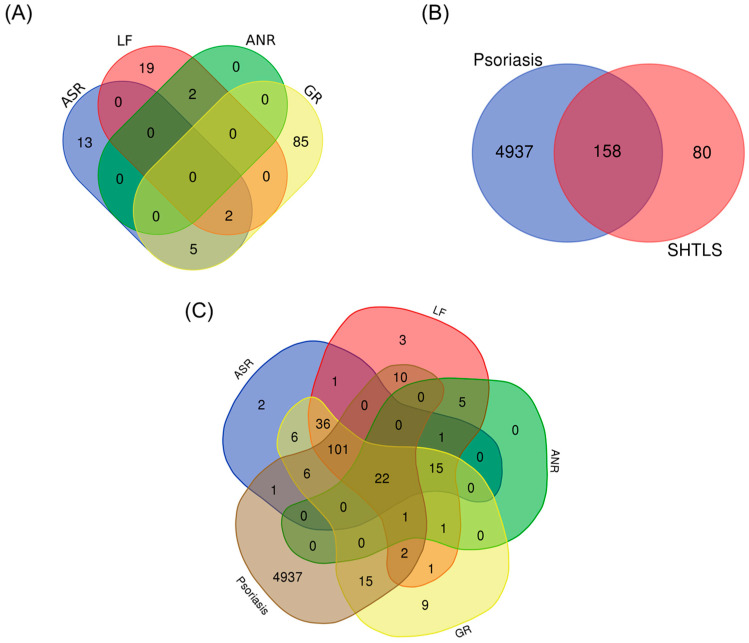
Venn diagram illustrating the constituent ingredients and their targets of SHTLS and psoriasis. (**A**) Distribution of bioactive compounds in the herbs comprising SHTLS. (**B**) Common target genes between SHTLS and psoriasis. (**C**) Distribution of target genes of the herbs comprising SHTLS and psoriasis.

The active compounds of the herbs comprising SHTLS were obtained from the traditional Chinese medicine systems pharmacology database and analysis platform (TCMSP) database and visualized as a Venn diagram (Figure 1A). Among the herbs, GR contained the highest number of bioactive compounds, followed by LF, ASR, and ANR. The significant compounds (i.e., those with at least one known target) of SHTLS and their corresponding herbs are listed in Supplementary Table S1. Representative marker compounds include luteolin (LF), liquiritin (GR), foromentin (ASR), and decursin (ANR), which have been widely studied for their anti-inflammatory and immunomodulatory properties. A total of 238 genes were associated with SHTLS, while 5095 genes were related to psoriasis. Further screening of SHTLS targets relevant to psoriasis identified 158 genes (Figure 1B). The distribution of all 5175 genes, categorized by their herbal origin, is depicted in a Venn diagram (Figure 1C).

### 2.2. Analysis of Protein–Protein Interaction (PPI) Network, Biological Process (BP), and Kyoto Encyclopedia of Genes and Genomes (KEGG) Enrichment of Key Target Genes of Sinhyotaklisan (SHTLS)

**Figure 2:**
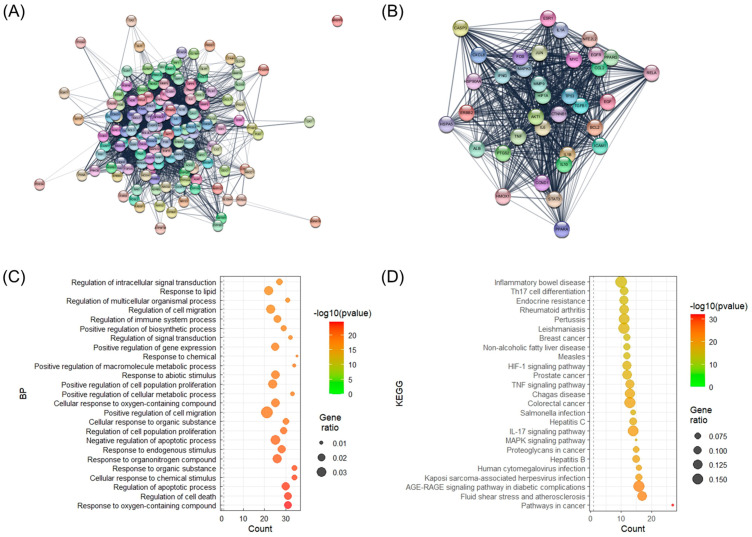
Illustration of the PPI network of target genes and BP enrichment analysis of their constituent genes. (**A**) PPI network of all target genes of SHTLS. (**B**) PPI network of key target genes of SHTLS. (**C**) Bubble plots displaying BP enrichment. (**D**) Bubble plots displaying KEGG enrichment.

A total of 158 SHTLS genes associated with psoriasis were analyzed within a protein–protein interaction (PPI) network to isolate key target genes based on network parameters (Figure 2A). As a result, 36 genes that exhibited above-average values for both network parameters were identified and collected (Table 1). The PPI interaction network of these 36 key genes was then visualized (Figure 2B). Among them, AKT1, TNF, IL6, TP53, ALB, and IL1B were identified as the most crucial target genes for the therapeutic effect of SHTLS on psoriasis. Biological process (BP) term enrichment analysis revealed that these key genes were highly associated with “Response to oxygen-containing compound”, “Regulation of cell death”, and “Regulation of apoptotic process” (Figure 2C). Furthermore, Kyoto Encyclopedia of Genes and Genomes (KEGG) pathway enrichment analysis identified four psoriasis-related pathways, including MAPK signaling pathway (ranked 8th), IL-17 signaling pathway (ranked 9th), TNF signaling pathway (ranked 14th), and HIF-1 (hypoxia-inducible factor 1) signaling pathway (ranked 16th) (Figure 2D).

### 2.3. Illustration of Key Target Genes in Psoriasis-Related Kyoto Encyclopedia of Genes and Genomes (KEGG) Pathways

**Figure 3:**
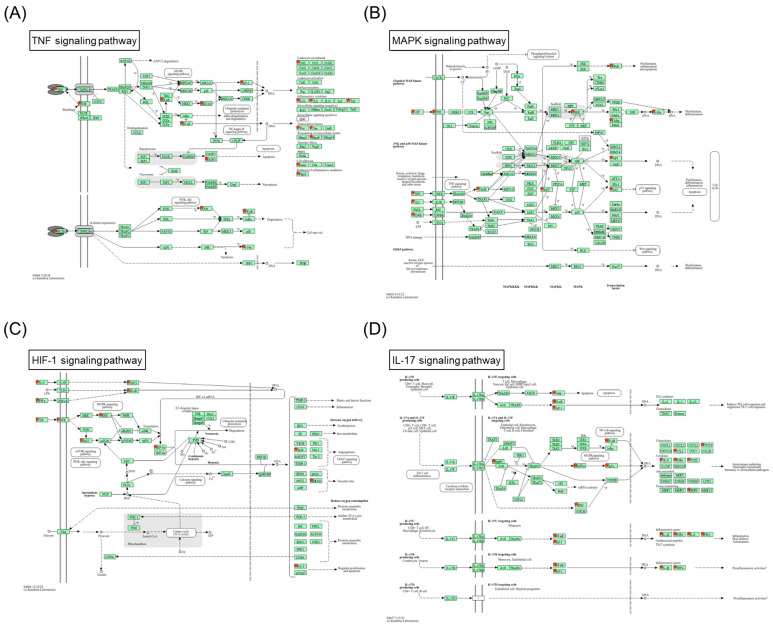
Predicted mechanism of action of SHTLS in major KEGG pathways associated with psoriasis. (**A**) TNF signaling pathway. (**B**) MAPK signaling pathway. (**C**) HIF-1 signaling pathway. (**D**) IL-17 signaling pathway.

To provide an integrative understanding of the mechanism of SHTLS in psoriasis, the key target genes involved in the four psoriasis-related KEGG pathways were marked in pathway illustrations (Figure 3A–D). As shown in Figure 3A, SHTLS appears to regulate the apoptotic cell death pathway triggered by TNF-α stimulation. In particular, it demonstrates potential modulation of the MAPK and NF-κB pathways, which mediate inflammatory responses activated by TNF-α and other pro-inflammatory cytokines (Figure 3B). Additionally, the HIF-1 signaling pathway is activated by hypoxia-inducible factors, including IL-6, interferon gamma (IFN-γ), and various growth factors, which may be regulated by SHTLS (Figure 3C). Notably, the VEGF signaling pathway is induced by this signaling pathway (Figure 3C, right side). Interestingly, psoriasis is recognized as an autoimmune skin disease, and the IL-17 signaling pathway, a major regulator of autoimmune diseases, has been found to be closely linked to the therapeutic mechanism of SHTLS (Figure 3D).

### 2.4. Visualization of the Herb-Compound-Target-Pathway Network and Enriched Biological Process (BP) Terms Network of Key Targets

**Figure 4:**
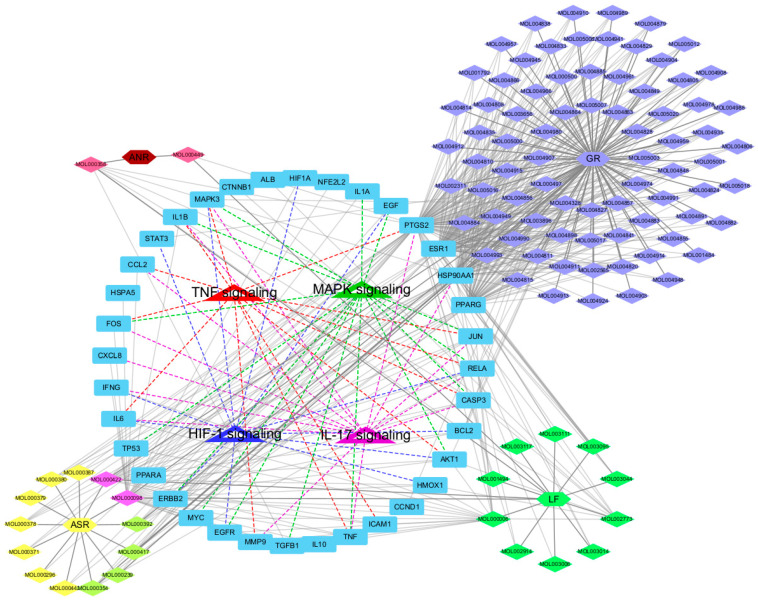
Illustration of the herb-compound-target-pathway network of SHTLS depicting its effect on psoriasis.

A multi-modal network integrating herbs, bioactive compounds, target genes, and pathways of SHTLS was constructed using Cytoscape (Figure 4). The complete network consisted of 147 nodes and 561 edges. At the center of the network, the four psoriasis-related pathways were connected to many of the key target genes, with 26 out of 36 genes connected to these pathways. Each compound was visually distinguished by color, indicating its herbal origin. Notably, GR contained the highest number of known bioactive ingredients (represented in Lavender Blue). Among the key target genes, MAPK3 and RELA were connected to all four psoriasis-related pathways, highlighting their critical role in the therapeutic mechanism of SHTLS. Additionally, IL1B, FOS, IL-6, TNF, AKT1, CASP3, and JUN were connected to three of the four pathways, further supporting their significance.

**Figure 5:**
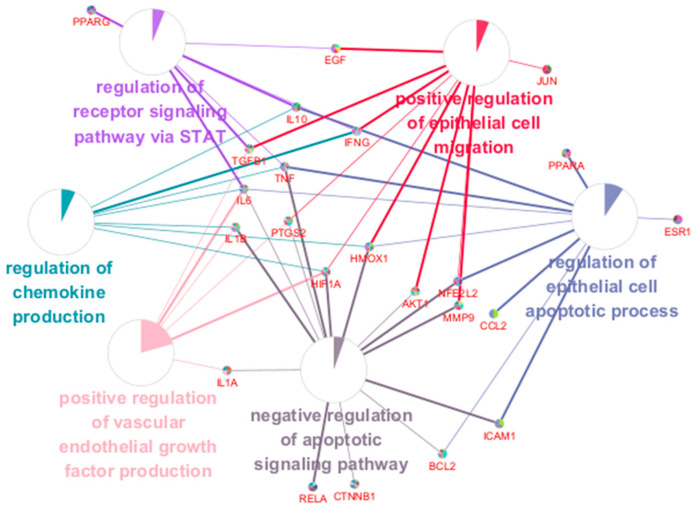
ClueGO BP enrichment analysis of key targets of SHTLS. A network displaying interactions between significant BP terms and their associated genes.

Using the core target list of SHTLS, a functional network of major pathways and their associated target genes was visualized using ClueGO in Cytoscape (Figure 5). This ClueGO network enabled the identification of BPs based on shared target genes. The key BP terms identified included: regulation of chemokine production, regulation of epithelial cell apoptotic process, regulation of receptor signaling pathway via STAT, positive regulation of VEGF production, negative regulation of apoptotic signaling pathway, and positive regulation of epithelial cell migration. Notably, HIF1A, IL-6, TNF, and IFNG were co-modulated by more than four BPs, indicating their central role in the mechanism of action of SHTLS in psoriasis treatment.

### 2.5. Effect of Sinhyotaklisan (SHTLS) Treatment on HaCaT Keratinocytes Viability

**Figure 6:**
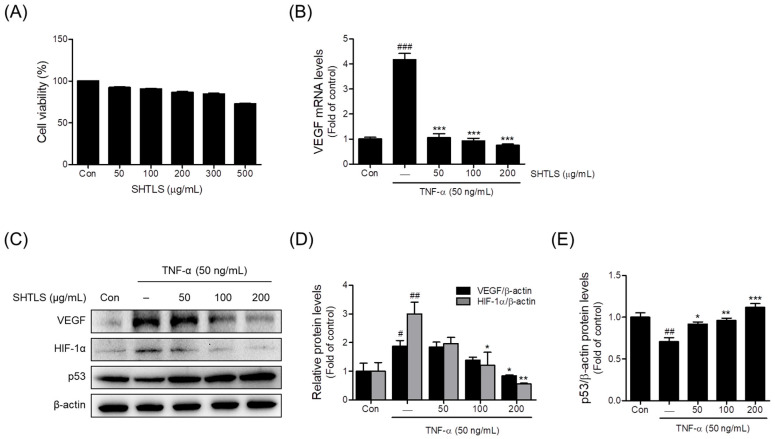
Effects of SHTLS on cell viability and angiogenesis markers in HaCaT cells. (**A**) Effect of SHTLS on HaCaT cell viability. (**B**) Effect of SHTLS on VEGF mRNA expression. (**C**) Effect of SHTLS on angiogenesis markers induced by TNF-α. (**D**) Quantification of relative VEGF and HIF-1α protein levels. (**E**) Quantification of relative p53 protein levels. Data are expressed as mean ± SEM. Significance is shown as ^#^ *p* < 0.05, ^##^ *p* < 0.01, and ^###^ *p* < 0.001 versus control, and * *p* < 0.05, ** *p* < 0.01, and *** *p* < 0.001 versus TNF-α-activated.

The cytotoxicity of SHTLS treatment at concentrations ranging from 50 to 500 μg/mL was evaluated in HaCaT keratinocytes. As shown in Figure 6A, the SHTLS treatment resulted in a gradual decrease in cell viability in a dose-dependent manner, with viability remaining relatively high (≥86.6%) at concentrations up to 200 μg/mL. However, at 300 μg/mL, viability decreased to 84.6 ± 0.9%, and a more pronounced reduction was observed at 500 μg/mL (72.7 ± 0.8%). To minimize cytotoxicity while ensuring reliable experimental conditions, subsequent experiments were conducted using concentrations up to 200 μg/mL.

### 2.6. Anti-Angiogenic Effect of Sinhyotaklisan (SHTLS) in Tumor Necrosis Factor-α (TNF-α)-Stimulated HaCaT Keratinocytes

TNF-α is known to trigger inflammation and promote angiogenesis, both of which are key features of psoriasis pathology [18,20,21]. To evaluate the anti-angiogenic effects of SHTLS, a TNF-α-induced psoriasis model in HaCaT cells was used. First, the expression of VEGF was assessed to determine how SHTLS modulates factors involved in angiogenesis within the psoriatic microenvironment. SHTLS pretreatment (50, 100, and 200 μg/mL) significantly downregulated the TNF-α (50 ng/mL)-induced elevation of VEGF mRNA expression in HaCaT keratinocytes, with an 81.7% reduction observed at 200 μg/mL (Figure 6B). VEGF protein expression was also dose-dependently reduced by SHTLS pretreatment (1 h), with significant inhibition observed at 200 μg/mL, with a 54.99% reduction (Figure 6C,D). Furthermore, TNF-α-induced HIF-1α protein levels were dose-dependently decreased by SHTLS pretreatment, with significance at 100 and 200 μg/mL, with an 81.40% reduction at 200 μg/mL (Figure 6C,D). In contrast, p53 levels, which were downregulated by TNF-α stimulation, were significantly upregulated by SHTLS pretreatment at all concentrations, with a 58.29% increase at 200 μg/mL (Figure 6C,E).

### 2.7. Anti-Proliferative Effect of Sinhyotaklisan (SHTLS) in Tumor Necrosis Factor-α (TNF-α)-Stimulated HaCaT Keratinocytes

**Figure 7:**
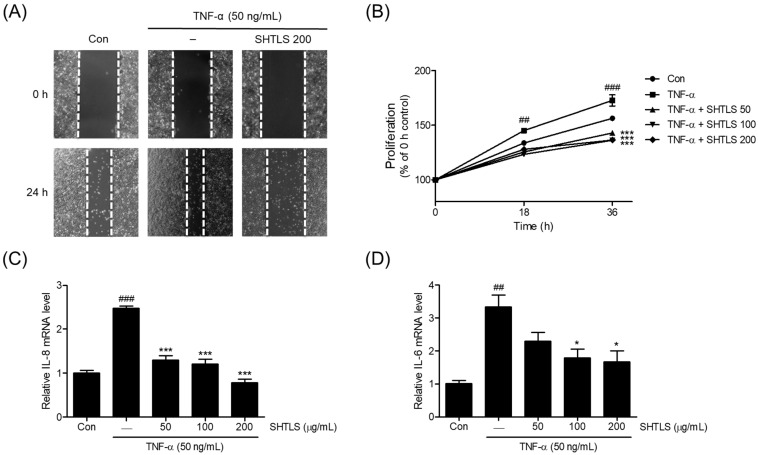
The effects of SHTLS on hyper-proliferative HaCaT keratinocytes within the TNF-α stimulation. (**A**) Scratch assay was performed to assess HaCaT keratinocytes proliferation (images captured at 40× magnification). (**B**) The proliferation rate of HaCaT cells cultured with SHTLS (50, 100, and 200 μg/mL) or TNF-α (50 ng/mL) was monitored. Data represent the percentage of the 0 h control. IL-8 (**C**) and IL-6 (**D**) mRNA expression was measured by qRT-PCR. HaCaT cells were pretreated with various concentrations of SHTLS for 12 h and then stimulated with TNF-α (50 ng/mL) for 1 h. The data are presented as fold change relative to the untreated control. Data are expressed as mean ± SEM. Significance is shown as ^##^ *p* < 0.01 and ^###^ *p* < 0.001 versus control, and * *p* < 0.05 and *** *p* < 0.001 versus TNF-α-activated.

The pro-inflammatory cytokine TNF-α has been reported to upregulate proliferation in conjunction with NF-κB activation in keratinocytes [14]. Thus, TNF-α was used to induce psoriasis-like hyperkeratosis in HaCaT cells. As shown in Figure 7A, TNF-α (50 ng/mL) increased the migration and proliferation of HaCaT cells, rapidly narrowing the scratched lesion. However, pretreatment with SHTLS (200 μg/mL) decreased migration, resulting in a wider gap under microscopic observation.

The proliferation of HaCaT cells was assessed using a 3-[4,5-dimethylthiazole-2-yl]-2,5-diphenyltetrazolium bromide (MTT) assay at 18 h and 36 h. TNF-α (50 ng/mL) treatment led to a significant increase in cell proliferation at both time points (18 h, *p* < 0.01; 36 h, *p* < 0.001). However, co-treatment with SHTLS significantly reduced proliferation, even in the presence of TNF-α stimulation, with notable decreases observed at both 18 h and 36 h (*p* < 0.001) (Figure 7B). These results suggest that SHTLS effectively inhibits TNF-α-induced keratinocyte proliferation.

Next, the mRNA expression of key pro-inflammatory cytokines, IL-8 and IL-6, involved in cell proliferation, was examined. As shown in Figure 7C, the IL-8 mRNA level increased upon TNF-α stimulation but was significantly downregulated by SHTLS pretreatment in a dose-dependent manner, with a 68.10% reduction at 200 μg/mL. Similarly, the IL-6 mRNA level was modulated by SHTLS, showing a significant reduction at concentrations of 100 and 200 μg/mL, with a 50.25% reduction at 200 μg/mL, in a manner similar to IL-8 expression (Figure 7D). These results indicate that the anti-proliferative effect of SHTLS against TNF-α stimulation is feasible within the viability range (≤200 μg/mL) of human keratinocytes.

### 2.8. Modulation of NF-κB and Mitogen-Activated Protein Kinase (MAPK) Signaling Pathways by Sinhyotaklisan (SHTLS) in Tumor Necrosis Factor-α (TNF-α)-Stimulated HaCaT Keratinocytes

**Figure 8:**
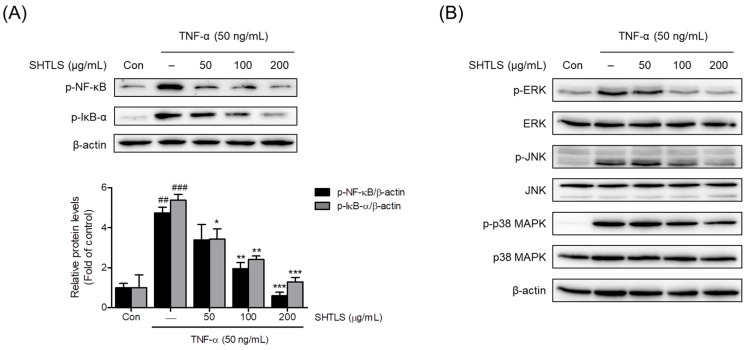
Effect of SHTLS on the NF-κB and MAPK pathways as demonstrated by Western blot analysis. (**A**) Effects of SHTLS on NF-κB pathway activation in TNF-α-stimulated HaCaT cells. Representative immunoblot images showing p-NF-κB, p-IκB-α, and β-actin in the upper panel, with quantification of relative p-NF-κB and p-IκB-α protein levels in the lower panel. β-actin was included as a loading control. (**B**) Effects of SHTLS on MAPK signaling pathway activation in TNF-α-stimulated HaCaT cells. Representative immunoblot images of phosphorylated and non-phosphorylated ERK, JNK, and p38 MAPK are presented. Data are expressed as mean ± SEM. Significance is shown as ^##^ *p* < 0.01 and ^###^ *p* < 0.001 versus control, and * *p* < 0.05, ** *p* < 0.01, and *** *p* < 0.001 versus TNF-α-activated.

Among the intracellular signaling pathways, the NF-κB and MAPK pathways are known to play crucial roles in psoriasis [30,31,32]. In particular, phosphorylated NF-κB levels are elevated in psoriasis, highlighting the importance of NF-κB signaling in disease progression [30,31,32]. Thus, the change in the relative phosphorylation ratio of NF-κB and IκB-α proteins following SHTLS treatment was analyzed using immunoblotting. The results showed that the phosphorylation ratios of NF-κB and IκB-α were increased by TNF-α (50 ng/mL) but were dose-dependently inhibited by SHTLS pretreatment, with statistically significant reductions at 100 and 200 μg/mL for p-NF-κB, with an 87.48% reduction at 200 μg/mL and at 50, 100, and 200 μg/mL for p-IκB-α, with a 76.03% reduction at 200 μg/mL (Figure 8A). These findings suggest that SHTLS inhibits the NF-κB signaling pathway in TNF-α-stimulated human keratinocytes, which may contribute to its effects.

The inhibitory effect of SHTLS on the MAPK pathway was examined through immunoblot analysis by comparing relative phosphorylation levels. As shown in Figure 8B, phosphorylation of extracellular signal-regulated kinase (ERK), c-Jun N-terminal kinase (JNK), and p38 MAPK was notably increased by TNF-α stimulation. However, SHTLS treatment dose-dependently suppressed the phosphorylation of all three MAPK markers. Immunoblot images showed that among the MAPK family, the ERK pathway was strongly modulated by SHTLS. These results suggest that the anti-angiogenic, anti-inflammatory, and anti-hyperproliferative effects of SHTLS are linked to the efficient inhibition of the MAPK signaling pathway.

### 2.9. Anti-Psoriatic Effect of Sinhyotaklisan (SHTLS) in an Imiquimod (IMQ)-Induced Psoriasis-like Mouse Model

**Figure 9:**
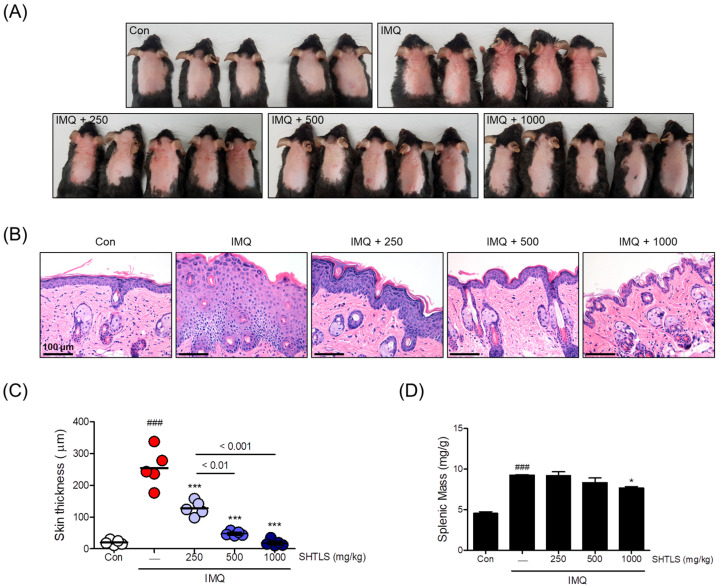
Effects of SHTLS on IMQ-induced psoriatic lesions in C57BL/6 mice. (**A**) Phenotype of dorsal skin from IMQ-treated mice showing the effects of SHTLS administration. (**B**) Histological analysis of dorsal skin lesions. Microscopic observation of H&E-stained dorsal skin tissue. Scale bar = 100 μm. (**C**) Epidermal thickness of dorsal skin was measured. (**D**) Splenic mass was measured and presented as spleen weight divided by body weight (mg/g). Five mice were used for the in vivo study and analysis. Data are expressed as mean ± SEM. Significance is shown as ^###^ *p* < 0.001 versus control, and * *p* < 0.05 and *** *p* < 0.001 versus IMQ-treated.

To evaluate the anti-psoriatic effects of SHTLS in vivo, skin tissues were examined for clinical and histological features after sacrifice. As shown in Figure 9A, mice in the IMQ group developed psoriasis-like skin lesions, including scaling, erythema, and thickening on the dorsal skin. However, mice administered SHTLS (250, 500, and 1000 mg/kg) showed an improvement in these clinical changes compared with the IMQ group in a dose-dependent manner. Pathological changes in the dorsal skin lesions were evaluated using H&E (hematoxylin and eosin) staining. Dermal thickness increased following IMQ cream application but was attenuated by SHTLS treatment in a dose-dependent manner, with a 92.80% reduction at 1000 mg/kg (Figure 9B,C). Furthermore, to assess the systemic immunomodulatory effect of SHTLS in the IMQ-induced psoriasis mouse model, spleens were harvested, and their weight was measured and normalized to body weight. As shown in Figure 9D, the IMQ group exhibited apparent splenomegaly, while the SHTLS-treated groups showed a tendency toward decreased splenic mass, particularly at the high dose of SHTLS (1000 mg/kg), with a 20.03% reduction, which was statistically significant (*p* < 0.05). These results suggest that SHTLS may alleviate psoriasis-like lesions and modulate the immune response.

## 3. Discussion

Current drugs prescribed to patients with psoriasis are required to address multiple pathological features in lesions. Major pathological changes include hyperproliferation of keratinocytes, increased dermal vascularity (angiogenesis), and immune cell infiltration in the lesion [33]. Meanwhile, the development of clinical anti-psoriatic therapeutics is currently focused on two different types of entities [34]. Several therapeutic biologics are under development for targeted therapy with different mechanisms of action. A recently approved example of this is bimekizumab, an antibody therapeutic that blocks the action of both IL-17A and IL-17F, which demonstrated favorable response and tolerance in phase 3 trial [35].

The second approach involves the development of orally available small-molecule drugs, which can enter cells and regulate various cellular mechanisms. Drugs in this category include Janus kinase (JAK) inhibitors, phosphodiesterase 4 (PDE4) inhibitors, and aryl hydrocarbon receptor agonist [34]. In addition to these conventional therapeutics, there is growing demand for the development of new medicines with pleiotropic, multi-target strategies for the management of psoriasis [27,36]. Numerous candidates for psoriasis treatment have been suggested in the field of traditional herbal medicine, targeting the major pathways mentioned above while comprising multiple active compounds.

As stated in an extensive review on the roles of cytokines involved in psoriasis, the pathophysiological characteristics of psoriasis reveal that numerous cytokines function as part of complex networks [37]. Among these cytokines, TNF-α plays a leading role in inducing keratinocyte proliferation, neutrophil influx, and vascular angiogenesis and has been established as a major pathogenic therapeutic target [34]. While mimicking the complex pathological features of psoriasis is challenging, and several models have been developed to study psoriasis in vitro [38], TNF-α alone was used in our study to induce pathological mechanisms in an in vitro environment. The IMQ-induced in vivo psoriasis model closely resembles the pathological characteristics of human psoriasis, exhibiting hyperkeratosis, T-cell/neutrophil infiltration, vascularization, and inflammatory cytokine expression, making it a suitable model for testing the efficacy of drug candidates [39].

As we reported previously, SHTLS showed favorable effects in attenuating the inflammatory response in lipopolysaccharide (LPS)-activated macrophages [26]. SHTLS significantly reduced the expression of COX-2, iNOS (inducible nitric oxide synthase), MAPK, NF-κB, and the antioxidant enzyme HO-1 in murine macrophages. These mechanisms of SHTLS were initially inferred based on its traditional use and the known pharmacological properties of its constituent ingredients. However, its potential pathways and mechanisms remain largely unstudied without any prior assumptions. By adopting a network pharmacology approach, we identified highly relevant targets and pathways from the intricate multi-target networks derived from the pharmaceutical profile of SHTLS. The TNF-α, MAPK, HIF-1α, and IL-17 signaling pathways were highly involved in the PPI network of 36 key targets of SHTLS, which are commonly recognized as major pathological features in the development of psoriasis (Figure 3 and Figure 4). Moreover, when these key targets were analyzed based on their BP terms, several pathological changes associated with psoriasis were implicated to be modulated by SHTLS, including “epithelial cell migration”, “chemokine production”, and “positive regulation of vascular endothelial growth factor production” (Figure 5).

Psoriasis is driven by multiple interacting immune signaling cascades. The IL-23 and IL-17 signaling axis plays a central role in psoriasis pathogenesis by driving the hyperproliferation and aberrant differentiation of epidermal keratinocytes, with IL-23 sustaining IL-17 cytokine production by pathogenic T cells. Moreover, as a critical intracellular kinase within this pathway, tyrosine kinase 2 mediates IL-23 receptor signaling and downstream STAT3 activation, making it a promising therapeutic target for disrupting the IL-23/IL-17-driven inflammatory cascade in psoriasis [40]. In addition, IL-1 and IL-36 cytokines contribute to keratinocyte hyperproliferation, impair epidermal differentiation, and sustain inflammatory signaling through the recruitment and activation of neutrophils and Th17 cells [41]. These effects are further enhanced by the synergistic interaction between IL-17 and TNF-α, which together contribute to immune dysregulation and the amplification of psoriatic inflammation [42]. Taken together, these cytokine-mediated pathways reflect fundamental pathological mechanisms of psoriasis. As demonstrated by our network pharmacologic analysis, SHTLS may exert therapeutic effects by modulating these key signaling cascades.

TNF-α is a crucial cytokine that mediates immune responses and apoptosis, maintained at a low level in healthy skin tissues [43]. According to previous studies, TNF-α, an inflammatory cytokine, correlates with other pro-inflammatory mediators and triggers the production of IL-6, IL-8, and VEGF [44]. Additionally, SHTLS has demonstrated anti-inflammatory properties and has been shown to effectively modulate TNF-α production in macrophages [26]. Therefore, we designated TNF-α (50 ng/mL) as the inducer of a psoriasis-like state in keratinocytes.

Upregulation of IL-8 by TNF-α stimulation has been reported to mediate cell proliferation by increasing matrix metalloproteinase (MMP) production in endothelial cells and HaCaT human keratinocytes [45]. The MMP family is involved in the breakdown of the extracellular matrix in normal skin physiology and plays major roles in angiogenesis, wound healing, and cell migration [46]. Specifically, among the MMP family, MMP-2 promotes the survival of keratinocytes, whereas MMP-9 stimulates terminal differentiation [47]. Furthermore, IL-8 triggers the activation of the NF-κB pathway [48]. Phosphorylation of NF-κB is regarded as an essential marker of psoriatic pathology, as it modulates cell proliferation [45,49]. In our study, SHTLS seems to exert a potential inhibitory effect on TNF-α-induced hyperproliferation, possibly through its regulation of NF-κB signaling activity (Figure 8A).

Another pathological feature of psoriasis is altered angiogenesis, with VEGF contributing significantly to the angiogenic process [18,19]. Psoriatic lesions show elevated VEGF level, suggesting that VEGF/VEGF receptor-targeted strategies could modulate psoriasis-related pathological changes [50]. Meanwhile, hypoxic conditions promote the stabilization of the HIF-1α, leading to an increase in HIF-1 activity [51]. HIF-1α is primarily activated in a hypoxic tumor microenvironment [52]. The increased expression of HIF-1α enhances VEGF production, thereby promoting angiogenesis [53]. Similarly, the skin epidermis is prone to HIF-1α overexpression in psoriatic lesions, leading to increased VEGF production [51,54,55]. Furthermore, the crosstalk between HIF-1α and p53, a key tumor suppressor protein, has been suggested in numerous studies [56,57]. P53 inhibits HIF-1 activity by inducing the ubiquitination and proteasomal degradation of HIF-1α, whereas the loss of p53 enhances HIF-1-dependent VEGF generation [58]. By modulating key pathological mechanisms of psoriasis, including the downregulation of HIF-1α and the restoration of p53 expression, SHTLS appears to significantly impact the crosstalk between these two critical markers, thereby contributing to its therapeutic potential in psoriatic lesions (Figure 6C–E).

Finally, we examined the systemic effectiveness of SHTLS in an in vivo model mimicking psoriasis. Based on previous studies using herbal extracts and considering the traditional safety of SHTLS components, we selected a dose range of 250, 500, and 1000 mg/kg to explore its therapeutic efficacy across multiple levels. These doses correspond to a human equivalent dose of approximately 20.25–81 mg/kg/day, which is within the range commonly considered acceptable for clinical herbal treatments [59]. A series of results, including the attenuation of dorsal skin lesions, reduction in skin thickness, and histological observations, suggest the potential of SHTLS in clinical studies. Splenomegaly often indicates an increased systemic immune response and inflammation [60]. Reduced spleen size in the SHTLS-treated group tentatively suggests a systemic alteration in immune response, and the key markers observed in our in vitro study might explain the underlying mechanism.

Our data collectively indicate that SHTLS exerts multi-level effects on psoriasis pathology—including suppression of keratinocyte hyperproliferation, angiogenesis, and inflammatory cytokine production—through the coordinated regulation of MAPK, NF-κB, and HIF-1α signaling. Compared to single-target therapies such as IL-17 inhibitors or PDE4 inhibitors, the multi-target nature of SHTLS, as inferred from the network pharmacology and confirmed through in vitro/in vivo assays, supports its potential as a broad-spectrum therapeutic candidate for psoriasis.

This study has several limitations. The absence of a reference drug for efficacy comparison is a significant limitation of this study. Another limitation is that we were unable to identify the specific active compounds responsible for the therapeutic effects of SHTLS. Moreover, as suggested by the findings of the network pharmacology analysis, further investigation is warranted to examine the effects of SHTLS on IL-17 signaling in more refined experimental models.

In conclusion, SHTLS appears to have significant potential as a treatment for psoriasis, as its mechanisms targeting core psoriasis-related pathways were identified through the network pharmacological analysis. SHTLS significantly reduced psoriatic changes and key markers in TNF-α-induced HaCaT cells. Regarding the two main characteristics of psoriasis, anti-angiogenic and anti-hyperproliferative effects were observed in HaCaT keratinocytes. The mechanism of SHTLS was further supported by its modulation of the MAPK and NF-κB signaling pathways. Finally, SHTLS demonstrated favorable effects against psoriatic skin lesions in a mouse model, indicating its potential as a novel candidate for psoriasis management. Nonetheless, it is important to note that this is a preliminary study and requires further validation in advanced models.

## 4. Materials and Methods

### 4.1. Acquisition of Active Ingredients and Targets Using Online Databases

TCMSP (https://old.tcmsp-e.com/tcmsp.php, accessed on 28 October 2024) [61] was used as a repository to collect information about the ingredients and targets of SHTLS. Potential bioactive ingredients in each herb were screened based on ADME (absorption, distribution, metabolism, and excretion) properties, particularly drug-likeness (DL, ≥0.18) and oral bioavailability (OB, ≥30%).

Relevant proteins targeted by each ingredient, as obtained from TCMSP, were validated and converted to official gene symbols using the GeneCards web database (https://www.genecards.org/, accessed on 1 November 2024) and STRING database (https://string-db.org/, accessed on 1 November 2024). Lists of ingredients and targets were sorted and uploaded as groups for each herb to the Bioinformatics and Evolutionary Genomics website (http://bioinformatics.psb.ugent.be/webtools/Venn/, accessed on 2 November 2024) to generate Venn diagrams and tables of genes or compounds.

Psoriasis-related target genes of SHTLS were identified by searching for “psoriasis” on GeneCards and extracting overlapping target genes of SHTLS from the resulting list of human genes. The network parameters of the PPI network comprising these genes were analyzed and ranked based on their degree and betweenness centrality (BC) scores. Target genes with values above the average degree and BC score were selected and designated as core target genes.

### 4.2. Construction of a Protein–Protein Interaction (PPI) Network and a Compound-Target-Pathway Network

The PPI network for core target genes was visualized by submitting the gene list to the STRING database, which generated the corresponding interaction network. The organism was set to “Homo sapiens” and default settings were used for constructing PPI network interactions. Submitted gene names were authenticated by comparing them with GeneCards entries.

A herb-compound-target-pathway network was constructed and visualized to better understand the connectivity between components. Nodes represented herbs, compounds, targets, and pathways, while edges represented interactions. Different node attributes were distinguished by color.

### 4.3. Gene Ontology (GO) and Kyoto Encyclopedia of Genes and Genomes (KEGG) Pathway Enrichment Analyses

Enrichment analysis of Gene Ontology (GO) terms and KEGG pathways was performed using the key target gene list in the STRING database. The results were exported as tables and processed for visualization as bubble plots created using the ggplot2 R package (version 3.5.1). The top 25 significant BP and KEGG pathways were ranked and displayed in a bubble plot, showing the gene ratio, *p*-value, and gene count.

Illustrations of major KEGG pathways were obtained by submitting key target genes to the DAVID database, with the organism set to “Homo sapiens”. Key genes were highlighted in the pathway map (https://davidbioinformatics.nih.gov/, accessed on 1 November 2024).

### 4.4. ClueGO Biological Process (BP) Term Enrichment Analysis of Key Targets

The network of enriched BP terms related to key SHTLS targets was analyzed and visualized using the ClueGO plug-in (version 2.5.10) in Cytoscape software (version 3.10.1) [62]. Gene lists were directly uploaded, and a BP annotation network was generated using the following settings: organism—Homo sapiens, GO term fusion enabled, *p*-value < 0.0005, GO tree interval (min 7-max 8 levels), and GO term connectivity score (Kappa score) = 0.6. The network visualization of BP terms significantly related to psoriasis and their associated key target genes was captured and presented as a figure.

### 4.5. Chemicals and Reagents

MTT, sodium phosphate, and other reagents were purchased from Sigma-Aldrich (St. Louis, MO, USA). Human TNF-α was obtained from Enzynomics (Daejeon, Republic of Korea). PCR primers for VEGF, IL-6, and IL-8 were supplied by Macrogen (Seoul, Republic of Korea). Primary antibodies for ERK, JNK, p38 MAPK, p-ERK, p-JNK, p-p38 MAPK, p-NF-κB, and p-IκB-α, as well as secondary antibodies, were supplied by Cell Signaling Technologies (Danvers, MA, USA). Additional primary antibodies, including VEGF, HIF-1α, and p53, were purchased from Santa Cruz Biotechnology (Santa Cruz, CA, USA).

### 4.6. Preparation of Sinhyotaklisan (SHTLS) Extract

In this study, we used the same batch of SHTLS extract as in our previous paper [26], and the high-performance liquid chromatography (HPLC) analysis results were reported in that study. In that study, chlorogenic acid was identified as a major compound using HPLC (retention time 8.36 min; 14.654 mg/g), and the fingerprint chromatogram was used to confirm batch consistency. The preparation method for the SHTLS extract follows the procedure described in our earlier work. Briefly, a mixture of LF (36 g), ANR (36 g), ASR (24 g), and GR (12 g) was soaked in 900 mL of 30% ethanol and subjected to extraction at 80 °C for 4 h. The extract was filtered through Whatman filter paper, concentrated using a rotary vacuum evaporator (EYELA, Tokyo, Japan), and then freeze-dried. The resulting dried powder (25.5 g, yield: 23.6%) was stored at −20 °C.

### 4.7. Cell Culture

An immortalized human keratinocyte cell line (HaCaT) was obtained from the CLS Cell Lines Service (Eppelheim, Germany). The cells were maintained in DMEM (Dulbecco’s Modified Eagle’s Medium; WELGENE, Gyeongsan, Republic of Korea) supplemented with 10% fetal bovine serum (WELGENE) and 1% penicillin-streptomycin solution (Gibco BRL, Gaithersburg, MD, USA), and were cultured in 100 mm Petri dishes. Cells were incubated at 37 °C in a humidified incubator containing 5% CO_2_ and were subcultured at approximately 3-day intervals upon reaching ~70% confluence.

### 4.8. Cell Cytotoxicity

The effect of SHTLS on HaCaT cell viability was examined using an MTT assay. HaCaT cells were plated at 4 × 10^4^ cells/well in 96-well plates and cultured until they reached 80% confluence. Cells were then treated with different concentrations (0–500 μg/mL) of SHTLS in serum-free medium. After incubation for 24 h, cell viability was assessed following the manufacturer’s MTT assay protocol. The cytotoxicity assay was performed in triplicate to ensure the reproducibility and reliability of the results.

### 4.9. Cell Proliferation Assay

A scratch assay was performed to evaluate the effect of SHTLS on HaCaT cell proliferation. Cells were plated in 24-well plates and cultured until they reached full confluency. A uniform scratch was then manually created using a pipette tip. After rinsing with DPBS, the culture medium was replaced with fresh medium containing SHTLS (200 μg/mL) and/or TNF-α (50 ng/mL). After 24 h, the scratch width was observed under a microscope.

Additionally, HaCaT cells (1 × 10^4^ cells/well) were seeded in 96-well plates and treated with TNF-α (50 ng/mL) in the presence or absence of SHTLS at various concentrations (50, 100, and 200 μg/mL) in 100 μL DMEM. Cell proliferation was assessed using an MTT assay at 18 h and 36 h. The percentage of proliferating cells was compared to the untreated control.

### 4.10. Quantitative Real-Time Polymerase Chain Reaction (qRT-PCR)

HaCaT cells were pretreated with various concentrations of SHTLS (50, 100, and 200 μg/mL) for 12 h and stimulated with TNF-α (50 ng/mL) for 1 h. Total RNA was extracted from HaCaT cells using TRIzol reagent (Invitrogen, Carlsbad, CA, USA). The concentration of extracted RNA was measured, and 1 μg of RNA from each sample was reverse-transcribed into cDNA using the RevertAid First Strand cDNA Synthesis Kit (Thermo Fisher Scientific, Waltham, MA, USA). The synthesized cDNA was then amplified by LightCycler^®^ 96 real-time PCR (Roche, Basel, Switzerland). The mRNA expression levels of target genes were normalized to glyceraldehyde-3-phosphate dehydrogenase (GAPDH). The sequences of the primers used for qRT-PCR were as follows: IL-6 (Forward 5′-AAG CCA GAG CTG TGC AGA TGA GTA-3′, Reverse 5′-TGT CCT GCA GCC ACT GGT TC-3′), IL-8 (Forward 5′-ACA CTG CGC CAA CAC AGA AAT TA-3′, Reverse 5′-TTT GCT TGA AGT TTC ACT GGC ATC-3′), VEGF (Forward 5′-GAG CCT TGC CTT GCT GCT CTA C-3′, Reverse 5′-CAC CAG GGT CTC GAT TGG ATG-3′), GAPDH (Forward 5′-GCA CCG TCA AGG CTG AGA AC-3′, Reverse 5′-TGG TGA AGA CGC CAG TGG A-3′).

### 4.11. Western Blot Analysis

Proteins were extracted from HaCaT cells using *Thermo Scientific^TM^ RIPA Lysis and Extraction Buffer* (Thermo Fisher Scientific, Waltham, MA, USA), supplemented with 1× *Xpert* phosphatase and protease inhibitor cocktail solutions (GenDEPOT, Barker, TX, USA). The lysates were centrifuged at 13,200 rpm for 30 min at 4 °C. Protein concentrations in the supernatants were determined using a BCA protein assay kit (Thermo Fisher Scientific). Equal amounts of proteins (25 μg) were separated via SDS-PAGE (10–12%, depending on the protein size) at 100 V for 90 min and transferred to PVDF membranes at 100 V for 1 h. The membranes were then blocked with 5% BSA or non-fat dry milk in PBST for 2 h at room temperature and then incubated overnight at 4 °C with primary antibodies (1:1000 dilution) against ERK, JNK, p38 MAPK, p-ERK, p-JNK, p-p38 MAPK, p-NF-κB, p-IκB-α, VEGF, HIF-1α, p53, and β-actin, with gentle shaking. After washing, the membranes were incubated with HRP-conjugated secondary antibodies (1:3000–1:5000 dilution) for 2 h at room temperature. Following washing with PBST, the immunoblot bands were detected using a chemiluminescence substrate (Thermo Fisher Scientific) and visualized with a chemiluminescence imaging system (Fusion Solo 2M; Vilber Lourmat, Marne-la-Vallée, France). Protein band intensities were quantified using ImageJ software (version 1.52a; National Institutes of Health, Bethesda, MD, USA), and expression levels were normalized to β-actin.

### 4.12. Animals

Six-week-old female C57BL/6 mice were purchased from Orient Bio Inc. (Seongnam, Republic of Korea). The mice were acclimated for two weeks and housed in a semi-pathogen free facility with ad libitum access to food and water. All experimental procedures were approved by the Institutional Animal Care and Use Committee of Dongguk University (Approval No. IACUC-2019-014-1). All animals were randomly assigned to each group at the beginning of the study. The health status of the mice was monitored throughout the experiment by daily observation for signs of distress or abnormal behavior. In addition, body weight was measured weekly. No adverse events were observed during the study period.

### 4.13. Induction of Psoriasis-like Lesions and Efficacy Testing in C57BL/6 Mice

During the experiment, 5% IMQ cream (Aldara^TM^, 3M Health Care Ltd., Loughborough, UK) was used to induce psoriasis-like skin symptoms in mice [63,64]. At eight weeks old, the mice were shaved and randomly divided into five groups (n = 5/group):

- Control group: Petrolatum cream and PBS (vehicle).
- IMQ group: 5% IMQ cream and PBS (vehicle).
- SHTLS 250 group: 5% IMQ cream and SHTLS 250 mg/kg.
- SHTLS 500 group: 5% IMQ cream and SHTLS 500 mg/kg.
- SHTLS 1000 group: 5% IMQ cream and SHTLS 1000 mg/kg.

SHTLS was administered orally once daily for one week. Psoriasis-like symptoms were then induced by applying 62.5 mg of IMQ cream to the shaved dorsal skin for seven consecutive days while oral gavage of SHTLS was continued.

At the time of sacrifice, the spleens and dorsal skin tissues were collected. To isolate the dorsal skin, mice were euthanized by inhalation of isoflurane gas (Hana Pharmaceutical, Seoul, Republic of Korea), and the hair on the dorsal area was removed using an electric shaver. The dorsal skin was then carefully excised using sterile scissors and separated from the underlying connective tissues. Residual fat and muscle were gently removed by scraping with a scalpel. The cleaned skin samples were cut into small sections and fixed in 5% neutral-buffered formalin for subsequent histological analysis [65]. Histological analysis of skin tissue was performed using H&E staining, and epidermal thickness was measured based on stained tissue images. Histological evaluations and measurements of epidermal thickness were conducted in a blinded manner by an investigator who was unaware of the group assignments, to reduce assessment bias. The spleen weight was recorded and normalized to the body weight.

### 4.14. Statistical Analysis

All experiments were performed at least three times (n = 3), and data are expressed as the mean ± standard error of the mean (SEM). Statistical analyses were conducted using one-way analysis of variance (ANOVA) followed by Tukey’s post hoc test for multiple comparisons. Significance levels were set at *p* < 0.05. All statistical tests were performed using GraphPad Prism version 5.03 (GraphPad Software Inc., San Diego, CA, USA).

## Figures and Tables

**Table 1:** Network parameters of significant target genes constituting the PPI network of SHTLS.

Gene Symbol	Degree	Betweenness Centrality
AKT1	126	0.038798
TNF	125	0.036107
IL-6	122	0.028189
TP53	120	0.026442
ALB	119	0.034818
IL1B	115	0.023147
CASP3	110	0.012303
PTGS2	110	0.02313
ESR1	109	0.022367
JUN	109	0.015067
MMP9	108	0.016962
HIF1A	107	0.011356
BCL2	106	0.010885
EGFR	106	0.02012
STAT3	105	0.010239
PPARG	103	0.01913
MYC	100	0.013551
CTNNB1	97	0.010247
MAPK3	97	0.01432
TGFB1	95	0.00717
FOS	92	0.014389
IFNG	92	0.007102
CCL2	92	0.008057
HSP90AA1	90	0.01548
CCND1	89	0.007204
IL-10	88	0.007228
CXCL8 (IL8)	87	0.006118
ERBB2	86	0.007181
EGF	86	0.004684
ICAM1	82	0.013478
IL1A	82	0.006435
HMOX1	79	0.004625
RELA	77	0.005654
NFE2L2	73	0.005323
PPARA	70	0.007071
HSPA5	66	0.006927

## Data Availability

Dataset available on request from the authors.

## Supplementary Materials

The following supporting information can be downloaded at: https://www.mdpi.com/article/10.3390/ijms26115082/s1.

## Abbreviations

The following abbreviations are used in this manuscript:

ADME	Absorption, distribution, metabolism, and excretion
BC	Betweenness centrality
BP	Biological process
DL	Drug-likeness
DMEM	Dulbecco’s Modified Eagle’s Medium
ERK	Extracellular signal-regulated kinase
GAPDH	Glyceraldehyde-3-phosphate dehydrogenase
GO	Gene Ontology
H&E	Hematoxylin and eosin
HIF-1	Hypoxia-inducible factor 1
HO-1	Heme oxygenase-1
HPLC	High-performance liquid chromatography
IFN-γ	Interferon gamma
IL-6	Interleukin-6
IL-8	Interleukin-8
IMQ	Imiquimod
iNOS	Inducible nitric oxide synthase
JAK	Janus kinase
JNK	c-Jun N-terminal kinase
KEGG	Kyoto Encyclopedia of Genes and Genomes
LPS	Lipopolysaccharide
MAPK	Mitogen-activated protein kinase
MMP	Matrix metalloproteinase
MTT	3-[4,5-dimethylthiazole-2-yl]-2,5-diphenyltetrazolium bromide
NF-κB	Nuclear factor-kappa B
OB	Oral bioavailability
PASI	Psoriasis Area and Severity Index
PDE4	Phosphodiesterase 4
PPI	Protein–protein interaction
qRT-PCR	Quantitative real-time polymerase chain reaction
ROS	Reactive oxygen species
SEM	Standard error of the mean
SHTLS	Sinhyotaklisan
STAT	Signal transducer and activator of transcription
TCMSP	Traditional Chinese Medicine Systems Pharmacology Database and Analysis Platform
TNF-α	Tumor necrosis factor-α
VEGF	Vascular endothelial growth factor
