# Supplementary material for: Sinhyotaklisan as a Potential Therapeutic for Psoriasis: Network Pharmacology and Experimental Validation
Source: Int J Mol Sci. 2025 May 25;26(11):5082. doi: 10.3390/ijms26115082 (PMC12155304; doi:10.3390/ijms26115082)
Supplement: Supplementary file 1 [file ijms-26-05082-s001.zip › ijms-3623565-supplementary.pdf]

Supplementary Table S1. Profiles of potential active compounds constituting SHTLS.

Herb	Mol no.	Compound	Herb	Mol no.	Compound
GR	MOL005020	dehydroglyasperins C	GR	MOL004898	(E)-3-[3,4-dihydroxy-5-(3-methylbut-2-enyl)phenyl]-1-(2,4-
	MOL005018	Xambioona		MOL004891	shinpterocarpin
	MOL005017	Phaseol		MOL004885	licoisoflavanone
	MOL005016	Odoratin		MOL004884	Licoisoflavone B
	MOL005012	Licoagroisoflavone		MOL004883	Licoisoflavone
	MOL005008	Glycyrrhiza flavonol A		MOL004882	Licocoumarone
	MOL005007	Glyasperins M		MOL004879	Glycyrin
	MOL005003	Licoagrocarpin		MOL004866	2-(3,4-dihydroxyphenyl)-5,7-dihydroxy-6-(3-methylbut-2-
	MOL005001	Gancaonin H		MOL004864	5,7-dihydroxy-3-(4-methoxyphenyl)-8-(3-methylbut-2-enyl)chromone
	MOL005000	Gancaonin G		MOL004863	3-(3,4-dihydroxyphenyl)-5,7-dihydroxy-8-(3-methylbut-2-
	MOL000500	Vestitol		MOL004857	Gancaonin B
	MOL004993	8-prenylated eriodictyol		MOL004856	Gancaonin A
	MOL004991	7-Acetoxy-2-methylisoflavone		MOL004855	Licoricone
	MOL004990	7,2',4'-trihydroxy - 5-methoxy-3 - arylcoumarin		MOL004849	3-(2,4-dihydroxyphenyl)-8-(1,1-dimethylprop-2-enyl)-7-hydroxy-5-
	MOL004989	6-prenylated eriodictyol		MOL004848	licochalcone G
	MOL004988	Kanzonol F		MOL004841	Licochalcone B
	MOL004980	Inflacoumarin A		MOL004838	8-(6-hydroxy-2-benzofuranyl)-2,2-dimethyl-5-chromenol
	MOL004978	2-[(3R)-8,8-dimethyl-3,4-dihydro-2H-pyrano[6,5-f]chromen-3-yl]-5-		MOL004835	Glypallichalcone
	MOL004974	3'-Methoxyglabridin		MOL004833	Phaseolinisoflavan
	MOL000497	licochalcone a		MOL004829	Glepidotin B
	MOL004966	3'-Hydroxy-4'-O-Methylglabridin		MOL004828	Glepidotin A
	MOL004961	Quercetin der.		MOL004827	Semilicoisoflavone B

	MOL004959	1-Methoxyphaseollidin
	MOL004957	HMO
	MOL004949	Isolicoflavonol
	MOL004948	Isoglycyrol
	MOL004945	(2S)-7-hydroxy-2-(4-hydroxyphenyl)-8-(3-methylbut-2-enyl)chroman-4-one
	MOL004941	(2R)-7-hydroxy-2-(4-hydroxyphenyl)chroman-4-one
	MOL004935	Sigmoidin-B
	MOL004924	(-)-Medicocarpin
	MOL004915	Eurycarpin A
	MOL004914	1,3-dihydroxy-8,9-dimethoxy-6-benzofurano[3,2-c]chromenone
	MOL004913	1,3-dihydroxy-9-methoxy-6-benzofurano[3,2-c]chromenone
	MOL004912	Glabrone
	MOL004911	Glabrene
	MOL004910	Glabranin
	MOL004908	Glabridin
	MOL004907	Glyzaglabrin
	MOL004904	licopyranocoumarin
**ASR**	MOL000442	1,7-Dihydroxy-3,9-dimethoxy pterocarpene
	MOL000439	isomucronulatol-7,2'-di-O-glucosiole
	MOL000433	FA
	MOL000387	Bifendate
	MOL000380	(6aR,11aR)-9,10-dimethoxy-6a,11a-dihydro-6H-benzofurano[3,2-
	MOL000379	9,10-dimethoxypterocarpan-3-O-β-D-glucoside
	MOL004824	(2S)-6-(2,4-dihydroxyphenyl)-2-(2-hydroxypropan-2-yl)-4-methoxy-2,3-
	MOL004820	kanzonols W
	MOL004815	(E)-1-(2,4-dihydroxyphenyl)-3-(2,2-dimethylchromen-6-yl)prop-2-en-1-
	MOL004814	Isotrifoliol
	MOL004811	Glyasperin C
	MOL004810	glyasperin F
	MOL004808	glyasperin B
	MOL004806	euchrenone
	MOL004805	(2S)-2-[4-hydroxy-3-(3-methylbut-2-enyl)phenyl]-8,8-dimethyl-2,3-
	MOL004328	naringenin
	MOL003896	7-Methoxy-2-methyl isoflavone
	MOL003656	Lupiwighteone
	MOL002565	Medicarpin
	MOL002311	Glycyrol
	MOL001792	DFV
	MOL001484	Inermine
	MOL004903	liquiritin
**LF**	MOL000006	luteolin
	MOL003128	dinethylsecologanoside
	MOL003117	loniceracetalides B_qt
	MOL003111	Centauroside_qt
	MOL003095	5-hydroxy-7-methoxy-2-(3,4,5-trimethoxyphenyl)chromone
	MOL003044	Chryseriol

	MOL000378	7-O-methylisomucronulatol
	MOL000374	5'-hydroxyiso-muronulatol-2',5'-di-O-glucoside
	MOL000371	3,9-di-O-methylnissolin
	MOL000033	(3S,8S,9S,10R,13R,14S,17R)-10,13-dimethyl-17-[(2R,5S)-5-propan-2-
	MOL000296	hederagenin
**GR ASR**	MOL000417	Calycosin
	MOL000392	formononetin
	MOL000354	isorhamnetin
	MOL000239	Jaranol
	MOL000211	Mairin
**ANR LF**	MOL000449	Stigmasterol
	MOL000358	beta-sitosterol
	MOL003036	ZINC03978781
	MOL002773	beta-carotene
	MOL003014	secologanic dibutylacetal_qt
	MOL003006	(-)-(3R,8S,9R,9aS,10aS)-9-ethenyl-8-(beta-D-glucopyranosyloxy)-
	MOL002914	Eriodyctiol (flavanone)
	MOL001495	Ethyl linolenate
	MOL001494	Mandenol
**GR LF ASR**	MOL000098	quercetin
	MOL000422	kaempferol
